# Genome-Wide Identification, Structural, and Gene Expression Analysis of BRI1-EMS-Suppressor 1 Transcription Factor Family in *Cucumis sativus*

**DOI:** 10.3389/fgene.2020.583996

**Published:** 2020-10-06

**Authors:** Si Ma, Tingting Ji, Meiting Liang, Shihui Li, Yongqiang Tian, Lihong Gao

**Affiliations:** Beijing Key Laboratory of Growth and Developmental Regulation for Protected Vegetable Crops, College of Horticulture, China Agricultural University, Beijing, China

**Keywords:** *Cucumis sativus*, BRI1-EMS-suppressor 1, gene structure, phylogeny, *cis*-element, abiotic stress

## Abstract

BRASSINOSTEROID INSENSITIVE1-EMS-suppressor 1 (BES1) is an essential regulator downstream of brassinosteroid signaling and plays important roles in plant stress response, growth, and development. To date, the regulation mechanisms of BES1 transcription factors have been identified and elucidated in model plants Arabidopsis and rice. However, little information is available regarding the BES1 family in *Cucumis sativus*. Therefore, this study conducted a genome-wide analysis of BES1 genes in cucumber. In cucumber, a total of six CsBES1 genes were identified, and their phylogenetic relationships, gene structures, and *cis*-elements in promoters were studied. *CsBES1* genes were distributed on four of seven chromosomes. Gene structure analysis showed that the intron–exon model of *CsBES1* genes was conserved and the CsBES1 protein contained a DUF822-conserved motif. Promoter *cis*-element prediction showed that plenty of developmental and stress- and hormone-related elements have been found in promoter regions of *CsBES1* genes. Meanwhile, BES1 was divided into three groups (I, II, and III) on the basis of phylogenetic relationship analysis in six plant species. In addition, *CsBES1* gene expression patterns were confirmed by transcription database and qRT-PCR analysis; the results showed that the expression of *CsBES1* genes had not only tissue-specific expression but also different types of *CsBES1* isoform which might respond to specific plant stresses. In summary, genome-wide identification, phylogeny, gene structure, and expression profile analysis of *CsBES1* genes in cucumber provided a referable theoretical information for further functional study of *CsBES1* genes and further facilitated the molecular breeding of cucumber.

## Introduction

Brassinosteroids (BRs), as a class of steroid hormone, are distributed ubiquitously in the plant kingdom. BRs can be used as stress alleviators to protect plants from a variety of abiotic and biotic stresses, such as drought, osmotic, cold, heat, wounding, and pathogen attack (Marcos et al., 2015; Krishna et al., 2017; Yan et al., 2018). In addition, BRs have been shown to act as key components of multiple physiological and developmental processes such as vascular development, cell elongation, cell division, and leaf senescence (Guo et al., 2013; Tong et al., 2014; Unterholzner et al., 2015). Given this, extensive researches have been done to reveal the molecular mechanisms and potential application of BRs in plants. To date, great progress has been achieved in BR signal transduction by proteomic surveys and molecular genetics in Arabidopsis.

In the BR signal transduction process, BRs are perceived through plasma membrane receptor kinase BRASSINOSTEROID INSENSITIVE1 (BRI1) and the co-receptor BRI1-Associated Receptor Kinase 1 (BAK1; Kim and Wang, 2010; Kim et al., 2011). The activation of BRI1 leads to sequential phosphorylation or dephosphorylation reaction, including positive regulators BRI1-SUPPRESSOR 1 (BSU1; Mora-García et al., 2004), BR SIGNALING KINASE 1 (BSK1; Tang et al., 2008), and protein phosphatase 2A (PP2A; Tang et al., 2011), and negative regulators BRI1-KINASE INHIBITOR 1 (BKI1; Wang and Chory, 2006), BRASSINOSTEROID INSENSITIVE 2 (BIN2; Li et al., 2002), and the 14-3-3 proteins (Gampala et al., 2007). Finally, dephosphorylation brassinazole-resistant 1 (BZR1) or BRI1-EMS-suppressor 1 (BES1) are translocated into the nucleus to regulate thousands of BR-target genes (Wang et al., 2002; Yin et al., 2005; Sun et al., 2010).

Brassinazole-resistant 1 and BES1, as the essential regulators in the downstream of BR signaling, have been intensively investigated to reveal their roles in plant stress response, growth, and development (Yin et al., 2005; Ryu et al., 2010; Guo et al., 2013). BES1 and phytochrome-interacting factor 4 (PIF4) interaction promotes cell elongation and plant growth by binding to nearly 2000 common target genes and controlling a core transcription network (Oh et al., 2012). BZR1 acts as an important regulator mediating the trade-off between growth and immunity upon inducing the expression of several WRKY transcription factors (Lozano-Durán et al., 2013). bil1-1D/bzr1-1D is a gain-of-function mutant in which a point mutation causes the stabilization and accumulation of the BIL1/BZR1 protein in the nucleus. When overexpressed in *Lotus japonicus*, bil1-1D/bzr1-1D increases resistance against thrip feeding compared with the wild type (Miyaji et al., 2014). BES1 and BZR1 could involve in the regulation of glucosinolate biosynthesis through binding to glucosinolate biosynthetic genes by the conserved N-terminal DNA-binding domain or indirectly through MYB factors (Guo et al., 2013). Thus, BZR1 and BES1 likely play a critical role in determining the primary BR signaling outputs. Besides, BES1 also is considered to be involved in the strigolactone (SL) signaling pathway. BES1 could interact with more axillary growth locus 2 (MAX2), which is a key component in the SL signaling pathway, and also can be degraded as the direct substrate of MAX2 to regulate shoot branching (Wang et al., 2013). BZR1 interacts *in vitro* and *in vivo* with REPRESSOR OF ga1-3 (RGA), which belongs to a member of the DELLA family of transcriptional regulators, to control cell elongation and plant growth (Li et al., 2012).

Plenty of researches on the characterization and functions of BES1/BZR1 have been conducted in Arabidopsis and rice, and some genome-wide analysis of BES1/BZR1 in tomato, maize, and Chinese cabbage have been performed to explore its gene structural and functional diversity during evolution. However, there is still lack of research on the phylogenetic relationship and function of the *BES1/BZR1* gene family in cucumber. Cucumber (*Cucumis sativus* L.) is an economically significant vegetable crop around the world, as well as a model system for studying fresh fruit development not only due to its vascular bundle structure but also due to its diverse floral sex types (Tanurdzic and Banks, 2004). In this study, we comprehensively described the function of BES1 and systematically analyzed the relative complete profile of the BES1 gene family by using the bioinformatic method in cucumber plants. We identified the members and phylogenetic relationship of the *CsBES1* gene family; analyzed the gene structure and promoter elements; and carried out the expression pattern of *CsBES1* genes in different organs, under various abiotic, and biotic stresses by quantitative real-time PCR. In addition, these findings will provide an insight for understanding the biological function of CsBES1 and help for molecular breeding of cucumber in the future.

## Materials and Methods

### Plant Material, Growth Conditions, and Stress Treatments

Cucumber (*Cucumis sativus* L.) cultivar “*Xintaimici”* was used for all the experiments. Cucumber seedlings were grown in a controlled-environment growth chamber under normal management (23/28°C, 10/14 h, dark/light, and 70–80% humidity). Seedlings with four mature leaves were treated with 150 mM NaCl or 10% PEG 6000 or transferred to a growth chamber set at 6°C as cold treatment. Leaves and roots were sampled on days 0, 6, 12, 24 h, 2 day, 3 day, 6 day, and 9 day after cold treatment and at 0, 1, 3, 6, 9, 12, and 24 h after NaCl and PEG treatments, respectively. All samples included at least three biological replicates. All samples were harvested, immediately frozen in liquid nitrogen, and stored at -80°C for further study.

### Identification of the CsBES1 Gene Family in Cucumber

The BES1 genes and amino acid sequences of cucumber and melon were obtained in the Cucurbit Genomics Network^1^ (Huang et al., 2009). The hidden Markov model (HMM) profile of the BES1_N domain (PF05687) was downloaded from the Pfam database (Pfam 29.0)^2^ to identify BES1 genes in the cucumber genome using HMMER 3.0 software with an *E*-value cutoff of 1. Arabidopsis BES1 protein sequences^3^ were used as a query against the predicted cucumber proteome sequences to identify all cucumber BES1 proteins. The BES1 protein sequences of Arabidopsis, rice, maize, and tomato were searched from the Arabidopsis Information Resource (Huala et al., 2001), the Rice Genome Annotation Project^4^ (Kawahara et al., 2013), the Maize Genetics and Genomics Database^5^, and the Sol Genomics Network^6^, respectively, for sequence analysis.

### Phylogenetic Relationship, Sequence Alignment, and Gene Structure Analyses

To investigate the phylogenetic relationships of *CsBES1* genes, a total of 35 BES1 protein sequences were identified from six plant species including cucumber, Arabidopsis, melon, tomato, rice, and maize. Multiple sequence alignments of the 35 BES1 amino acid sequences were performed with ClustalW (Higgins et al., 1996) using default parameters. A phylogenetic tree, in which the degree of support for a particular grouping pattern was evaluated using bootstrap (1000 replicates) values, was constructed using the software MEGA 5.0 via the maximum-likelihood method (Tamura et al., 2011). The MEME server^7^ and Tbtools were employed to analyze the motif composition of BZR1/BES1 in cucumber and Arabidopsis. The protparam tool^8^ was use to analyze the physicochemical characteristics of deduced CsBES1 amino acid sequences, including theoretical molecular weight (MW), isoelectric point (pI), and grand average of hydropathicity (GRAVY). The Cell-PLoc 2.0^9^ was used to analyze the subcellular localization of 6 CsBES1 amino acid sequences.

### Gene Expression Analysis Upon the RNA-Seq Data

The transcription data of *BES1* genes from different tissues (BioProject Name: PRJNA80169; PRJNA271595; and PRJNA258122) and under biotic stresses (BioProject Name: PRJNA376073; PRJNA388584; and PRJNA292785) in cucumber were collected from the Cucurbit genomics expression database^10^. Hi-seq data of 10 cucumber tissues containing root, stem, leaf, male flower, female flower, ovary, expanded fertilized ovary (7 days after flowering), expanded unfertilized ovary (7 days after flowering), base of the tendril, and tendril were used for expression of *BES1*. We used the Reads Per Kilobase per Million mapped reads (RPKM) values of *BES1* genes and then plotted it using the Pheatmap package in R software. Three heat maps of *BES1* genes in different cucumber organs were obtained and three heat maps of *BES1* genes in response to gibberellic acid (GA), powdery mildew, and downy mildew.

### RNA Extraction and qRT-PCR

Total RNA was extracted from frozen leaves and roots using RNeasy Plant kit (Huayueyang, Beijing, China) according to the manufacturer’s protocol. cDNA was synthesized from 1000 ng RNA using FastKing cDNA Dispelling RT SuperMix Kit (TIANGEN, Beijing, China). cDNA was diluted 1:10 for further quantitative reverse transcription polymerase chain reaction (qRT-PCR). The qRT-PCR reactions for detecting gene expression by using SYBR Premix Ex Taq^TM^ (Tli RNase H Plus; TaKaRa Shuzo, Shiga, Japan) according to the manufacturer’s protocol. Relative gene expression was calculated using ΔΔCT values obtained from the formulas ΔCT = target CT – reference CT and ΔΔCT = treated sample ΔCT – untreated sample ΔCT/ΔΔCT = other organ ΔCT – root ΔCT, and the calculation of the gene expression levels followed the 2^–△^^△^^CT^ method described by Livak and Schmittgen (2001). All qRT-PCR reactions were conducted at at least three biological replications and three technical repeats. The cucumber Tublin gene (Csa4G000580) was used as the reference gene. The primers used for qRT-PCR are listed in Supplementary Table 1.

## Results

### Identification of the BES1 Gene Family in Cucumber and Phylogenetic Relationship Analysis

A total of six *BES1* genes in cucumber were identified by comprehensive analysis in CuGenDB (available online: http://cucurbitgenomics.org/) and PlantTFDB (available online: http://planttfdb.cbi.pku.edu.cn/index.php?sp=Csg). The annotation IDs of *BES1/BZR1* genes in cucumber were Csa1G467200, Csa2G361450, Csa4G083490, Csa4G056530, Csa6G003450, and Csa6G501930. *CsBES1s* were located on chromosomes 1, 2, 4, and 6. To better understand characteristics of *CsBES1/CsBZR1*, we carried out systematically the statistical analysis on the protein physicochemical indices (Table 1). The six *CsBES1/CsBZR1* genes varied greatly in ORF length from 936 bp (Csa2G361450) to 2097 bp (Csa6G003450), and their encoded proteins ranged from 311 (Csa2G361450) to 698 (Csa6G003450) amino acids. The molecular weight ranged from 34.19 kDa (Csa2G361450) to 78.35 kDa (Csa6G003450), and the isoelectric point varied from 5.78 (Csa6G003450) to 9.17 (Csa2G361450). In addition, the GRAVY ranged from −0.689 (Csa6G501930) to 0.715 (Csa2G361450), and the subcellular localization predicted that all of the six CsBES1/CsBZR1 were localized in nucleus by Plant-mPLoc 2.0^11^.

**TABLE 1:** The characteristics of BZR1/BES1 genes from cucumber.

**Genes**	**Locus name**	**Chr.**	**Location**	**Subcellular localization**	**ORF length**	**Protein physicochemical characteristics**			
						**Length (aa)**	**MW (KDa)**	**pI**	**GRAVY**
CsBES1-1	Csa1G467200.1	1	16768701∼16771593 (-)	N	984	327	35.35	8.97	–0.644
CsBES1-2	Csa2G361450.1	2	17199641∼17201363 (+)	N	936	311	34.19	9.17	0.715
CsBES1-3	Csa4G056530.1	4	4704832∼4711322 (+)	N	2010	669	75.26	5.91	–0.404
CsBES1-4	Csa4G083490.1	4	5606824∼5610735 (+)	N	978	325	34.68	8.50	–0.572
CsBES1-5	Csa6G003450.1	6	340459∼346769 (-)	N	2097	698	78.35	5.78	–0.441
CsBES1-6	Csa6G501930.1	6	25188710∼25191519 (+)	N	960	319	34.64	8.96	–0.689

*Abbreviation: Chr., chromosome; N, nucleus; Length (aa), length of amino acid; MW, molecular weight; pI, isoelectric point; and GRAVY, grand average of hydropathicity.*

**FIGURE 1:**
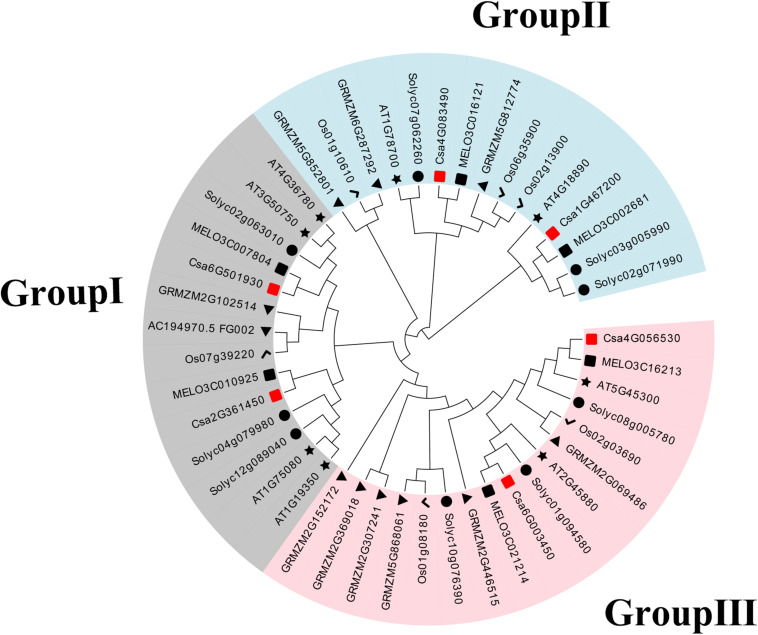
Phylogenetic relationships in BES1 proteins from cucumber (Cs), Arabidopsis (AT), melon (MELO), tomato (Soly), maize (GRMZM), and rice (Os). The neighbor-joining tree was generated including 6 BES1 proteins from cucumber, 8 from Arabidopsis, 6 from melon, 9 from tomato, 11 from maize, and 6 from rice.

To establish the functional and evolutionary relationship of the *CsBES1/CsBZR1* gene family, we constructed a phylogenetic tree using the amino acid sequences of six putative cucumber *BES1*, eight Arabidopsis *BES1*, six melon *BES1*, nine tomato *BES1*, six rice *BES1*, and eleven maize *BES1* (Table 2). Based on phylogenetic tree classification, all selected BES1 proteins were classified into three groups, in which Csa2G361450 and Csa6G501930, Csa1G467200 and Csa4G083490, and Csa4G056530 and Csa6G003450 belonged to group I, group II, and group III, respectively (Figure 1).

**TABLE 2:** Identified members of the BES1/BZR1 gene family in different plants.

**Species**	**Gene family (number)**
*Arabidopsis thaliana*	*AtBES1*, *BZR1* and *BEH1* (8)
*Cucumis sativus*	*CsBZR1*/*BES1* (6)
*Cucumis melo*	*CmBZR1*/*BES1* (6)
*Solanum lycopersicum*	*SlBZR1*/*BES1*/*LAT61* (9)
*Oryza sativa*	*OsBZR1* (6)
*Zea mays*	*ZmBES1*/*BZR1* (11)

### Gene Structure and Conserved Motif Analysis in Cucumber

**FIGURE 2:**
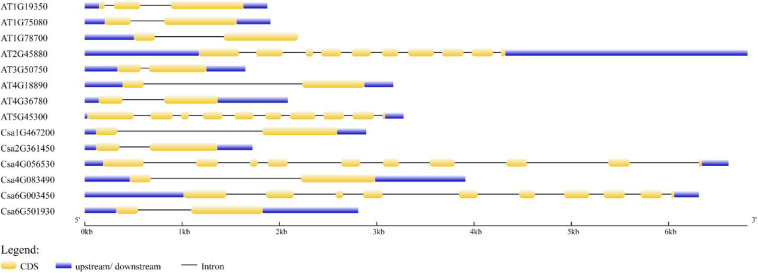
Exon and intron structures of BEs1 in *Cucumis sativus* and Arabidopsis thaliana. Exons are shown as yellow boxes; introns were shown as black lines. Upstream and downstream untranslated regions are shown as blue boxes. Scales are shown at the bottom of the diagram.

The distribution of exons and introns was the important information to understand gene structure. Thus, we analyzed the structural features of six *CsBES1* genes and eight *AtBES1* genes, including the number of exons and introns, and the length of exons, introns, and untranslated regions (Figure 2). The gene size varied from ∼1.5 kb in At3G50750 to 7.0 kb in At2G45880 and Csa4G056530. The structure analysis of *BES1/BZR1* genes from cucumber and Arabidopsis showed that there were three different types of structure distribution: the majority of *BES1/BZR1* genome contained two exons and one intron. However, only At1G19350 contained three exons and two introns, while At2G45880, At5G45300, Csa4G056530, and Csa6G003450 included ten exons and nine introns. Structural variation in *BES1* genes might be generated due to the gene duplication or incorporation after transcription during evolutionary history. These results suggested that different *BES1* genes may be functionally diversified.

**FIGURE 3:**
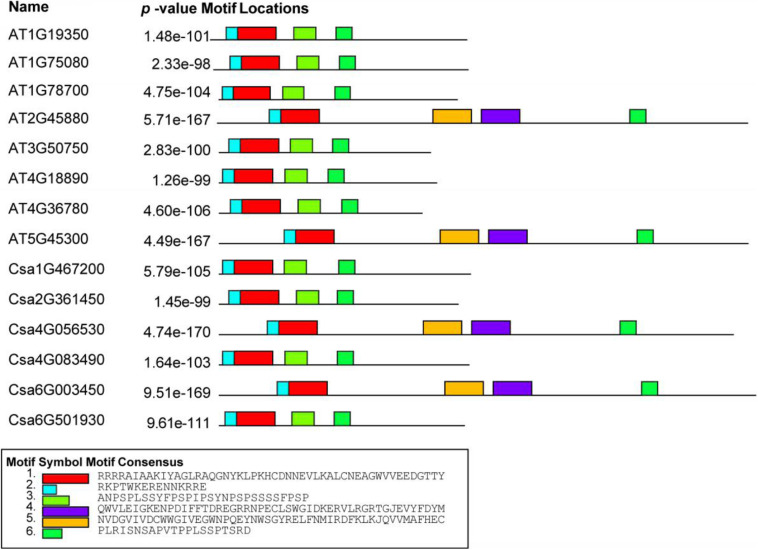
Conserved motif analysis of BES1 amino acid from Arabidopsis and *Cucumis sativus*. Motif analyses were conducted using MEME online software and Tbtools as described in the method. Different color boxes represent various types of conserved motifs. The gene IDs were listed on the left part of this figure. The amino acid sequences of key motifs are listed at the bottom of this figure.

To better understand the diversity and similarity of CsBES1 proteins, the conserved motifs were analyzed. As shown in Figure 3, a schematic overview of these conserved motifs was provided and demonstrated that all BES1 from cucumber and Arabidopsis included at least four conserved motifs located in N- and C-terminals. DUF822, also known as BES1_N (turquoise and red in Figure 3 and Supplementary Figure 1A), was found in all CsBES1 and AtBES1 protein sequences. Besides, two glycosylation-conserved sites (yellow and purple colors in Figure 3 and Supplementary Figure 1B) were found in At2G45880, At5G45300, Csa4G056530, and Csa6G003450.

### Analysis of *Cis*-Elements in the CsBES1 Promoters

**FIGURE 4:**
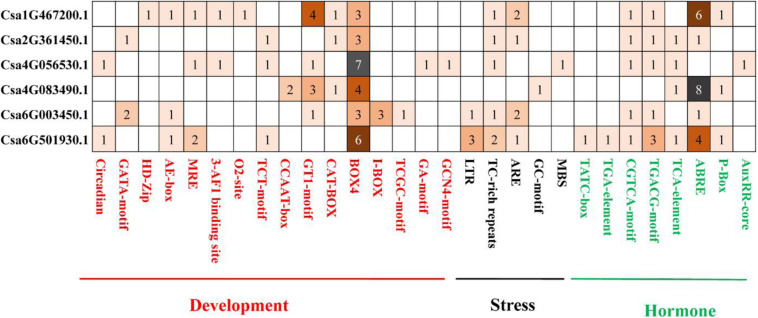
Distribution of development and stress- and hormone-related cis elements in cucumber BES1 promoters. ∼2.0-kb promoter sequences of six CsBES1 genes were predicted. The gene IDs were shown in the left part of this figure. The elements were shown at the bottom of this figure. The number in squares showed the number of elements in promoter regions.

To better understand the potential regulatory mechanisms of *CsBES1* genes during growth and development stages, under abiotic or biotic stresses and in response to different hormones, we identified the presence of *cis*-elements in the promoter regions of *CsBES1* genes. A 2.0-kb promoter region of all the *CsBES1* genes was scanned, and the *cis*-elements, which could possibly control the expression of *CsBES1*, were predicted using PLANTCARE tools. The results are shown in Figure 4.

For stress-related elements, **(1)** the ARE element was found in four selected promoter regions of Csa1G467200, Csa2G361450, Csa6G003450, and Csa6G501930, among which two ARE elements were identified in Csa1G467200 and Csa6G003450. **(2)** The TC-rich repeat element was found in five selected promoter regions of Csa1G467200, Csa2G361450, Csa4G056530, Csa6G003450, and Csa6G501930, and at least two TC-rich repeats elements were predicted in Csa6G501930. **(3)** The LTR element was only found in two promoter regions of Csa6G003450 and Csa6G501930. **(4)** The GC-motif and MBS elements were only found in Csa4G083490 and Csa4G056530, respectively. This suggested that *CsBES1* family genes played different roles in response to various stresses. For example, Csa6G003450 and Csa6G501930 with LTR elements might respond to low-temperature stress, while Csa4G056530 with MBS elements might respond to drought stress.

For hormone-related elements, **(1)** the ABRE element was found in five promoter regions of Csa1G467200, Csa2G361450, Csa4G083490, Csa6G003450, and Csa6G501930, of which at least four ABRE elements were found in Csa1G467200, Csa4G083490, and Csa6G501930. Since ABRE is an important ABA response element, *CsBES1* is probably involved in the ABA signal pathway. **(2)** The CGTCA-motif and TGACG-motif elements were found in five promoter regions of Csa1G467200, Csa2G361450, Csa4G056530, Csa6G003450, and Csa6G501930, implying that *CsBES1* may respond to jasmonic acid. **(3)** The TATC box and TGA element were found only in Csa6G501930, indicating that Csa6G501930 might be involved in the GA or auxin pathway.

For growth and development-related elements, **(1)** there were at least three BOX4 elements predicted in all six CsBES1 promoter regions. **(2)** The GT1 motif was found in promoter regions of Csa1G467200, Csa4G056530, Csa4G083490, and Csa6G003450. **(3)** The HD-Zip and O2-site elements were only found in the promoter region of Csa1G467200.

### Expression Patterns of BES1 in Cucumber Various Organs

**FIGURE 5:**
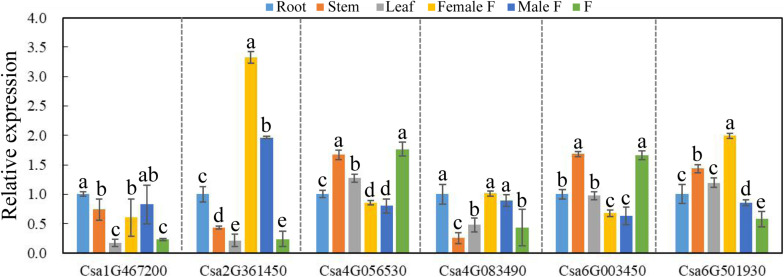
The expression pattern of CsBES1 genes in different cucumber organs. The Cucumber Tublin gene was the reference gene used to calibrate the relative gene expression. Error bars represent ± SE (*n* = 3). One-way analysis of variance was conducted by Duncan’s new multiple-range test, *n* = 3; different letters above the bars indicate significant differences (*P* < 0.05). Abbreviation: Female F, female flower; Male F, male flower; and F, fruit.

**FIGURE 6:**
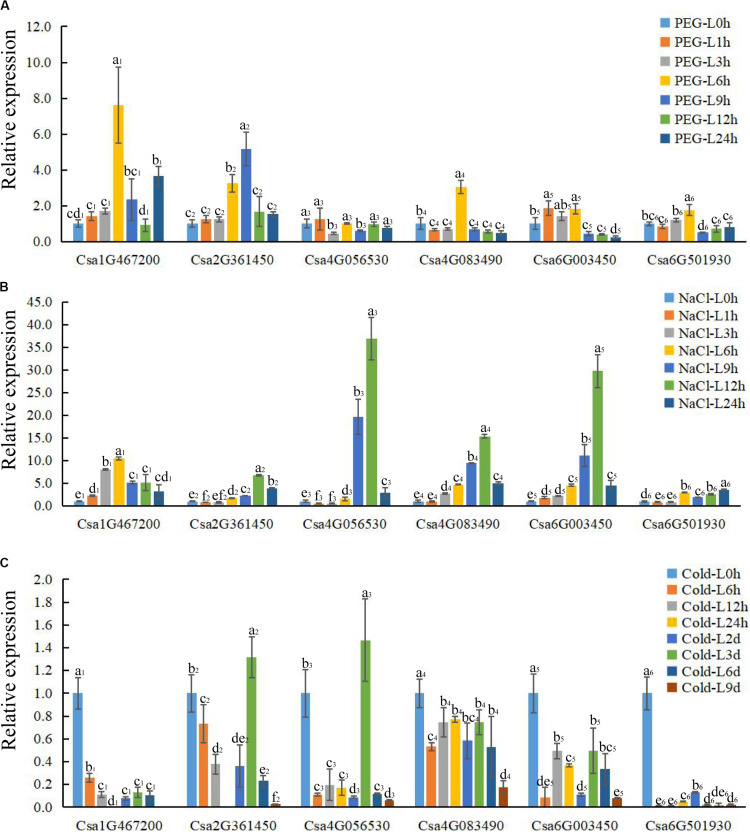
The expression profile of CsBES1 genes in cucumber leaves in response to different abiotic stresses. **(A)** Drought treatment (20% PEG6000); **(B)** salt treatment (150 mM NaCl); and **(C)** cold treatment (6°C). Error bars represent ± SE (*n* = 3). One-way analysis of variance was conducted by Duncan’s new multiple-range test, *n* = 3; different letters above the bars indicate significant differences (*P* < 0.05). Leaves were harvested at 0, 1, 3, 6, 9, 12, and 24 h after drought and salt treatments; the times of cold treatment were 0, 6, 12, 24 h, 2 day, 3 day, 6 day, and 9 day.

**FIGURE 7:**
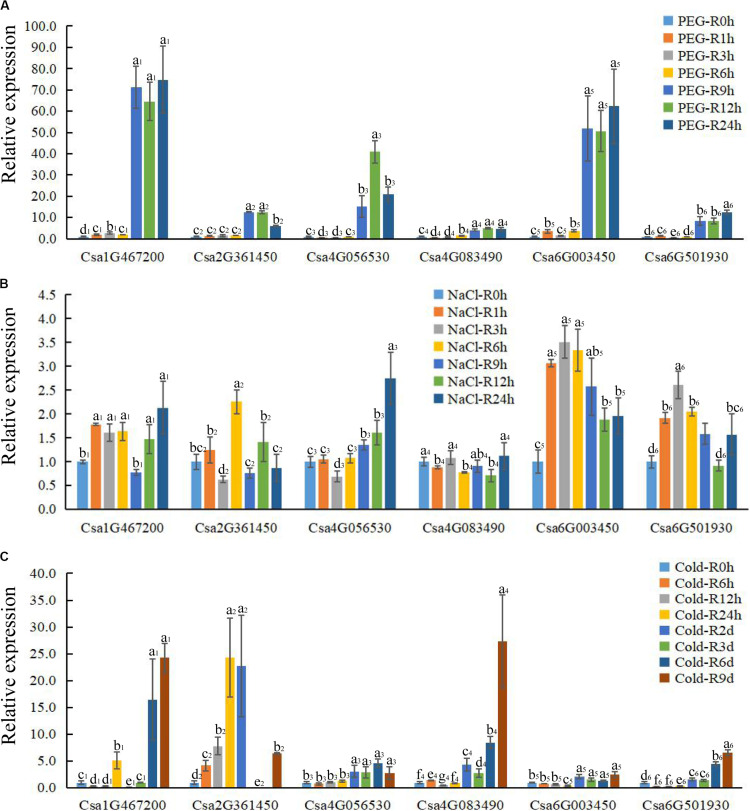
The expression profile of CsBES1 genes in cucumber roots in response to different abiotic stresses. **(A)** Drought treatment (20% PEG6000); **(B)** salt treatment (150 mM NaCl); and **(C)** cold treatment (6°C). Error bars represent ± SE (*n* = 3). One-way analysis of variance was conducted by Duncan’s new multiple-range test, *n* = 3; different letters above the bars indicate significant differences (*P* < 0.05). Roots were harvested at 0, 1, 3, 6, 9, 12, and 24 h after drought and salt treatments; times of cold treatment at 0, 6, 12, 24 h, 2 day, 3 day, 6 day, and 9 day.

To explore the possible functional roles of *CsBES1s* in developmental processes, the expression patterns of *CsBES1s* in root (R), stem (S), leaf (L), female flower (FF), male flower (MF), and fruit (F) were performed by qRT-PCR. As shown in Figure 5, Csa1G467200 and Csa2G361450 had a relatively low expression level in L and F, while Csa2G361450 showed the highest expression in flowers, especially in female flowers. Csa4G083490 was expressed highly in roots and fruits compared to its low expression in stems. Csa4G056530 and Csa6G003450 exhibited a similar expression pattern and had relatively high expression levels in stems and fruits compared with other tissues, and they did also respond similarly to some, although not all, of the abiotic stresses as analyzed in Figures 6, 7. Interestingly, it is notable that Csa4G056530 and Csa6G003450 were from Group III of the phylogenetic tree and shared highly similar and distinct gene structures (nine exons compared with two exons for all other BES1 genes) and the particular long glycosylation-conserved site (Figures 2, 3).

The RNA-sequencing database from the Cucurbit genomics database showed that the expression profiles of most *CsBES1*s were consistent with the results obtained by qRT-PCR except Csa2G361450. The Csa2G361450 transcription level showed the highest expression in flowers upon qRT-PCR data (Figure 5), but its expression was much higher in stems from the RNA-Seq database (Supplementary Figure 2). It is reasonable due to samples harvested in two different cucumber species and at different sampling times. Moreover, the expression profile of *CsBES1*s in different root development zones (differentiation, elongation, and meristematic zones) and fruit types (long and short fruits) were determined. The database indicated that Csa6G501930 was highly expressed in all tissues compared to other family genes, and in elongation zones and long fruits compared to other tissues (Supplementary Figures 2B,C). These results suggested the various expression patterns of each BES1 in different cucumber organs and indicated that they probably play different roles in cucumber different organs.

### Expression Pattern of BES1 Under Different Abiotic Stress Conditions

To elucidate the functions of *CsBES1*s in response to hormones and under different stresses (abiotic and biotic stresses), the expression profiles of *CsBES1*s in leaves and roots were conducted by qRT-PCR (Figures 6, 7) and the RNA-sequence database (Supplementary Figure 3). We tested the expression level of *CsBES1*s in leaves and roots at 0, 1, 3, 6, 9, 12, and 24 h after NaCl (150 mM) and drought (20% PEG 6000) treatments, and at 0, 6, 12, 24, 2 day, 3 day, 6 day, and 9 day after cold (6°C) treatment.

After drought treatment, the expression of Csa1G467200, Csa2G361450, and Csa4G083490 in leaves significantly increased at 6 h and then decreased compared to the control, while the expression of Csa6G003450 and Csa6G501930 kept a relatively stable level until 6 h and then decreased about 4 folds after 9 h of treatment. The expression of Csa4G056530 had no obvious change after treatment in leaves (Figure 6A). In roots, all the *CsBES1* genes had a sharp increase at 9 h to 24 h after treatment. In particular, Csa1G467200, Csa6G501930, and Csa6G003450 attached the peak point at 24 h, while Csa4G056530 and Csa4G083490 showed the highest point at 12 h after treatment (Figure 7A).

After NaCl treatment, Csa1G467200 showed an up-regulation between 8 and 10 folds at 3 h and 6 h after treatment compared to control in leaves; Csa2G361450 had a higher expression at 12 h and 24 h compared with control; Csa4G056530, Csa4G083490, and Csa6G003450 increased sharply at 9 h and 12 h after treatment; and Csa6G501930 exhibited a slight upregulation from 6 h to 24 h after treatment (Figure 6B). In roots, the expression of Csa1G467200, Csa6G501930, and Csa6G003450 increased steeply at 1 h after treatment; Csa2G361450 had a high expression at 6 h, and the Csa4G056530 expression attached the peak point at 24 h after treatment compared to the control; and Csa4G083490 showed no difference at all time points (Figure 7B).

After cold treatment, in leaves, Csa1G467200, Csa4G083490, Csa6G003450, and Csa6G501930 showed a decrease at all time points compared to the control; Csa2G361450 and Csa4G056530 showed the highest point at 3 day and then sharply decreased compared with control (Figure 6C). In roots, the expression of Csa4G056530, Csa4G083490, Csa6G003450, and Csa6G501930 was up-regulated at 2 day until to 9 day after treatment. The expression of Csa1G467200 and Csa2G361450 had similar changing patterns: increase–decrease–increase. Specifically, Csa1G467200 expressed an increase at 24 h, 6 day, and 9 day, while Csa2G361450 showed an increase at 6 h to 2 day and 9 day after treatment (Figure 7C).

In addition, we explored the expression pattern of *CsBES1* genes in leaves after GA treatment, downy mildew treatment, and powdery mildew treatment based on the RNA-sequence database. The results showed that Csa6G501930 had the highest expression at 6 h after GA treatment, and Csa2G361450 and Csa4G083490 had an increasing and decreasing trend after GA treatment, respectively, (Supplementary Figure 3A). The downy mildew and powdery mildew treatments lead to an overall decrease in Csa6G501930. Csa2G361450 was up-regulated at 6 h after downy mildew treatment, and Csa6G003450 showed an increase at 48 h after powdery mildew treatment (Supplementary Figure 3B).

## Discussion

The BES1 gene family has been previously reported in several plants, including Arabidopsis, tomato, Chinese cabbage, maize, and *Brassica napus* (Yin et al., 2005; Wu et al., 2016; Gao et al., 2018; Song et al., 2018; Yu et al., 2018; Cao et al., 2020). To date, the regulation mechanisms of BES1 transcription factors have been identified and elucidated in Arabidopsis and rice (Yin et al., 2005; Bai et al., 2012). However, little information is available regarding the BES1 family in cucumber. Therefore, this study conducted a genome-wide analysis of BES1 genes and proteins in cucumber. Based on phylogenetic relationship, conserved motifs, and gene structures analysis, it was found that there were six *CsBES1* genes distributed in three groups. Csa4G056530 and Csa6G003450, which belonged to group III, included ten exons and nine introns and possessed a particular long glycosylation conserved site. Accordingly, they had the longest amino acids and the largest molecular weight. By contrast, other four CsBES1s only included two exons and one intron. Specifically, Csa1G467200 and Csa4G083490 had a long segment of intron and were assigned to group II, while Csa2G361450 and Csa6G501930 contained a short segment of intron and were assigned to group I. Meanwhile, all of the CsBES1 proteins had one conserved DUF822 motif. These findings indicated that some intron (or motif) gain or loss events might be occurred during the evolution process in *CsBES1* genes and that various structures probably endowed these *CsBES1* genes with different functions.

The plant *cis*-element is an important molecular switch to regulate gene transcription during plant growth and development as well as in their responses to stresses and hormones. According to bioinformatics analysis, various *cis*-elements were found in *CsBES1* promoters, in which some gene specific *cis*-element was predicted. Notably, TATC-box (GA response element), and TGA-element (auxin response element) were only found in the Csa6G501930 promoter. This result was consistent with the transcription database, which showed that Csa6G501930 expression could be induced by GA (Supplementary Figure 3A). In the GA signaling pathway, RGA is a transcription factor belonging to the DELLA family protein. In Arabidopsis, the RGA protein could decrease the abundance and transcription level of AtBZR1. AtBZR1 (At1G75080) could interact with RGA *in vitro* and *in vivo* to participate the BR and GA signaling pathway (Li et al., 2012). In addition, AtBZR1 (At1G75080) also belonged to Group I similar to Csa6G501930 (Figure 1), and they had a similar gene structure (Figure 2) and conserved motif (Figure 3), which indicates that Csa6G501930 might participate in the brand GA signaling pathway as well as AtBZR1 (At1G75080). Auxin response factors (ARFs) are key transcription factors that regulate auxin-responsive gene expression. ARF6 and ARF7 have been identified to interact with BZR1 to coordinate in the growth and development of plants through the auxin and BR signaling pathway (Youn et al., 2016). Besides, many stress-related *cis*-elements were also predicted in *CsBES1* promoters, such as cold response element (LTR), and drought response element (MBS). In Arabidopsis, AtBES1 (At1G19350) was targeted for autophagy-induced degradation through directly interacting with DOMINANT SUPPRESSOR OF KAR 2 (DSK2) to respond abiotic stresses (Nolan et al., 2017). Together, these results provide referable evidence that CsBES1 may play important roles in the hormone regulation pathway and stress response in cucumber.

In this study, several experiments have been performed to characterize the expression profiles of CsBES1 genes in different cucumber organs in response to various abiotic stresses and hormones. It was noted that Csa2G361450 was highly expressed in flowers and showed a closer relationship with either At1G19350 or At1G75080 according to the phylogenetic tree (Figure 5). At1G75080 (BZR1) has been proven to be involved in photoperiodic flowering by regulating its target gene, BR ENHANCED EXPRESSION 1 (BEE), which directly binds to the FLOWERING LOCUTS T (FT) promoter to activate the transcription of FT and promote flowering initiation (Wang et al., 2019). Another study reported that AtBES1 (At1G19350) could directly bind to the promoter regions of *SPL/NZZ*, *TDF1*, *AMS*, *MS1*, and *MS2* genes, which are important transcription factors controlling anther and pollen development (Ye et al., 2010). These results suggested that Csa2G361450 probably participates in flower development. The transcription database showed that GA treatment induced an increase in the expression of Csa6G501930 and Csa2G361450 (Supplementary Figure 3A). BES1/BZR1, as a class of master transcription factor in the BR signaling pathway, mediated a direct cross talk between GA and other plant hormones (Li et al., 2018). In rice, OsBZR1 (Os07g39220) directly binds to the promoters of GA biosynthesis-related genes to regulate GA content for promoting cell elongation (Tong et al., 2014). Moreover, Csa6G501930 and Csa2G361450 had a close relationship in Group I based on phylogenetic tree analysis (Figure 1). This result suggested that Csa6G501930 and Csa2G361450 are probably involved in the GA signaling pathway.

In plants, BR has been universally recognized as an important phytohormone involved in a variety of stress responses (Krishna, 2003). Accordingly, BES1/BZR1, which control BR-regulated gene expression, has also been demonstrated to be critical in mediating plant responses to abiotic stresses including salt, drought, cold, heat, and freezing (Nolan et al., 2020). In this study, after drought treatment, Csa1G467200 expression was induced in both leaves and roots, while Csa4G056530 and Csa6G003450 were induced only in roots. Csa6G003450 expression showed a significant increase in leaves but not in roots after salt treatment. However, the expression of Csa4G083490 was decreased in leaves but increased in root after cold treatment. It was interesting that Csa1G467200, Csa6G003450, and Csa6G501930 were reduced in leaves but induced in roots after cold treatment (Figures 6, 7). These results revealed the different expression patterns of *CsBES1* genes under various abiotic stresses, thereby indicating that these genes may play different but essential roles in response to abiotic stresses.

## Conclusion

In this study, we systematically analyzed the structures, conserved motifs, and phylogenetic relationships of *CsBES1* genes and their expression profiles in different cucumber organs under various abiotic stresses. A total of six putative *CsBES1* genes were identified in cucumber. These six genes exhibited different expression patterns in cucumber organs and response to variously abiotic stresses. The comprehensive analysis on *CsBES1* was expected to provide basic information for further studying the functions of *CsBES1* and for the molecular breeding of cucumber.

## Data Availability Statement

All datasets presented in this study are included in the article/Supplementary Material.

## Author Contributions

LG, YT, and SM conceived and designed the experiments. SM, TJ, ML, and SL performed the experiments and collected the data. SM and TJ executed the data analyses. All authors contributed to the interpretation of the results. SM, LG, and YT wrote the drafts.

## Conflict of Interest

The authors declare that the research was conducted in the absence of any commercial or financial relationships that could be construed as a potential conflict of interest.

## Footnotes

1 http://cucurbitgenomics.org/organism/2

2 http://pfam.sanger.ac.uk/

3 http://www.arabidopsis.org

4 http://rice.plantbiology.msu.edu/index.shtml

5 https://www.maizegdb.org/

6 https://solgenomics.net/organism/

7 http://meme-suite.org/tools/meme

8 https://web.expasy.org/protparam/

9 http://www.csbio.sjtu.edu.cn/bioinf/Cell-PLoc-2/

10 http://cucurbitgenomics.org/rnaseq/home

11 http://www.csbio.sjtu.edu.cn/bioinf/Cell-PLoc-2/

## Supplementary Material

Supplementary Figure 1 Multiple alignments of BES1 domains from Arabidopsis and cucumber amino acid sequence.

Supplementary Figure 2 The expression profile of CsBES1 genes in different cucumber organs based on RNA-sequence database.

Supplementary Figure 3 Transcription data were used to analyze the expression profile of CsBES1 genes in cucumber leaves in response to different abiotic, biotic stresses and hormone.

Supplementary Table 1 Primers used in the study.
