# Supplementary figures and images for: Genome-Wide Identification, Structural, and Gene Expression Analysis of BRI1-EMS-Suppressor 1 Transcription Factor Family in Cucumis sativus
Source: Front Genet. 2020 Oct 6;11:583996. doi: 10.3389/fgene.2020.583996 (PMC7573293; doi:10.3389/fgene.2020.583996)

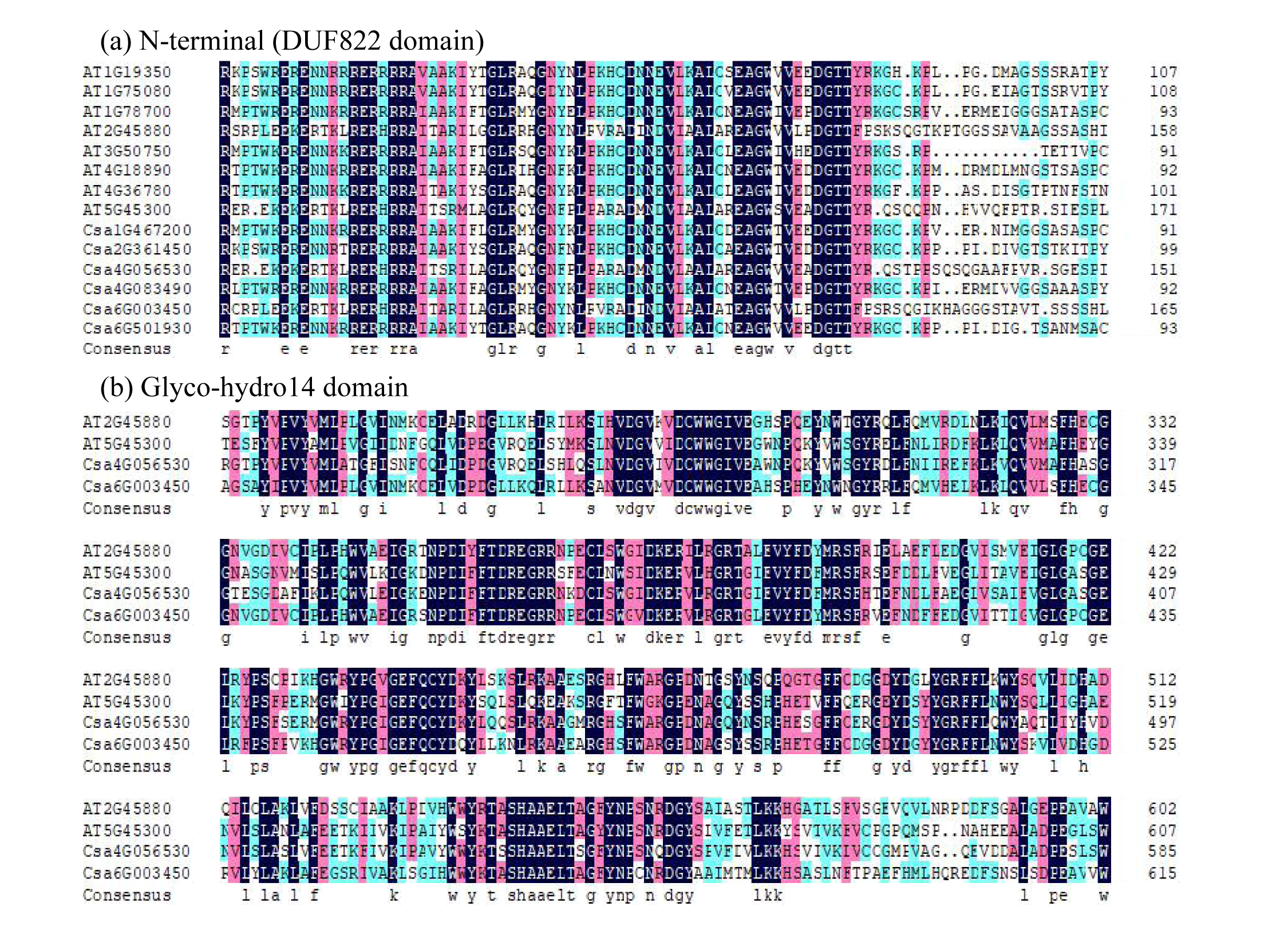

Supplement: Supplementary Figure 1 — Multiple alignments of BES1 domains from Arabidopsis and cucumber amino acid sequence. [file Image_1.TIF]

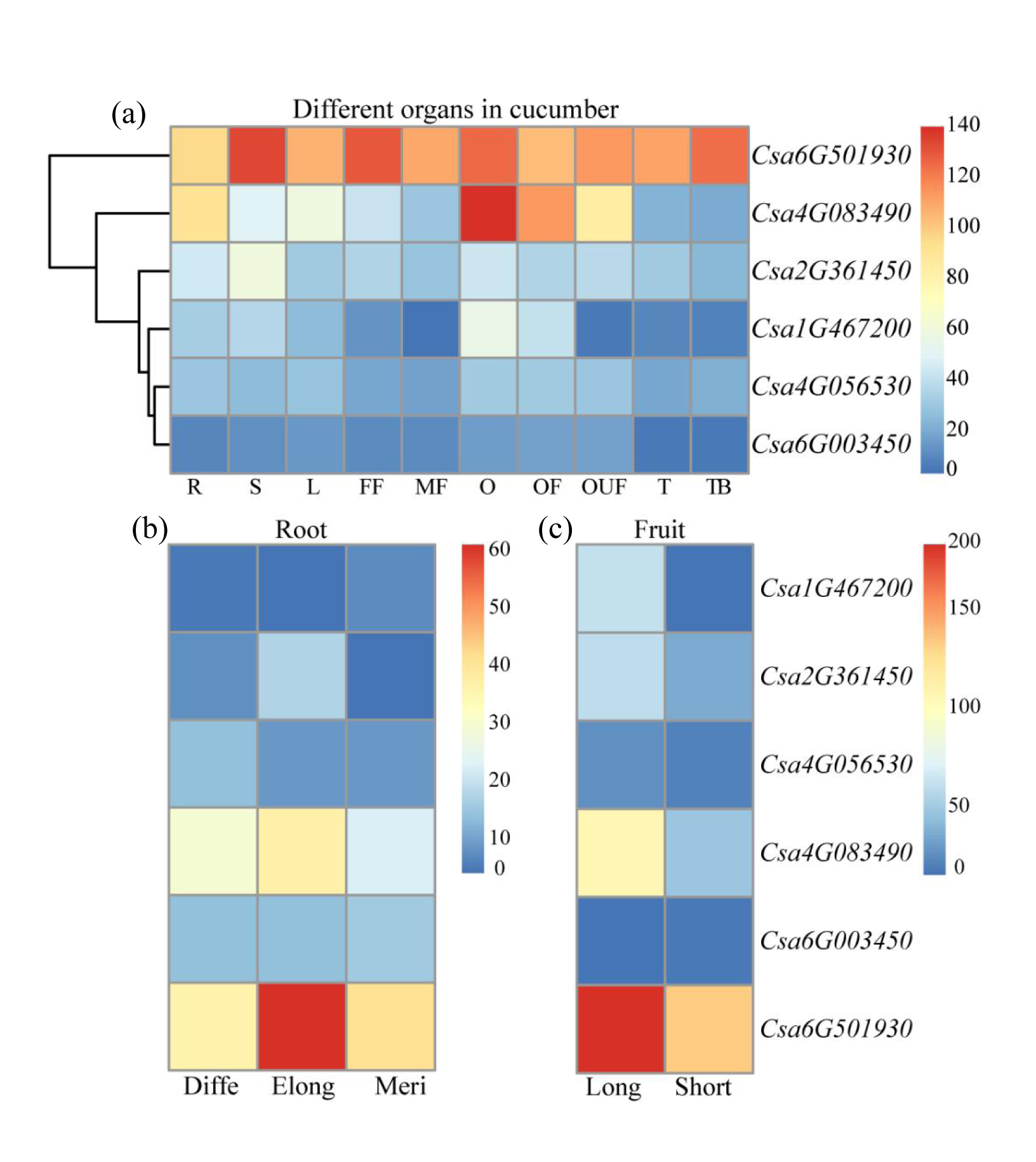

Supplement: Supplementary Figure 2 — The expression profile of CsBES1 genes in different cucumber organs based on RNA-sequence database. [file Image_2.TIF]

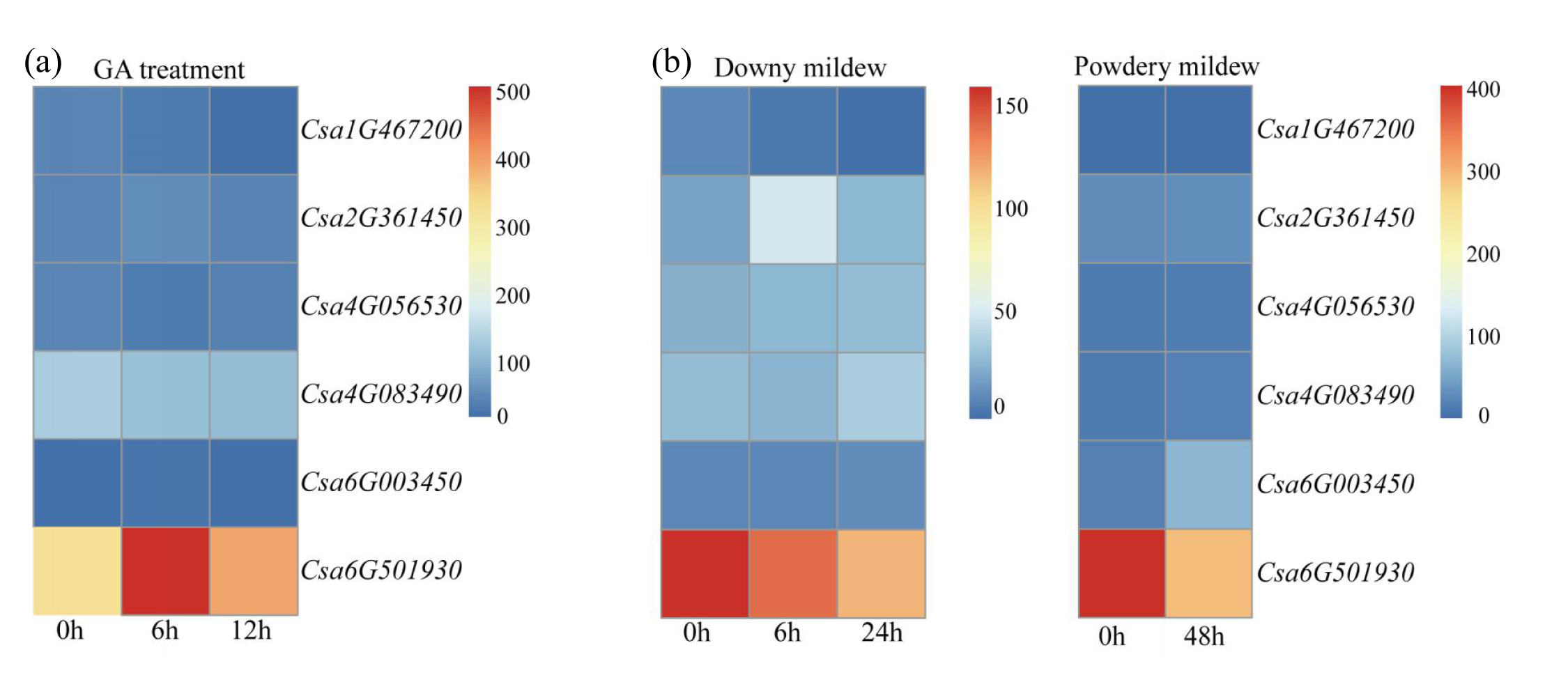

Supplement: Supplementary Figure 3 — Transcription data were used to analyze the expression profile of CsBES1 genes in cucumber leaves in response to different abiotic, biotic stresses and hormone. [file Image_3.TIF]

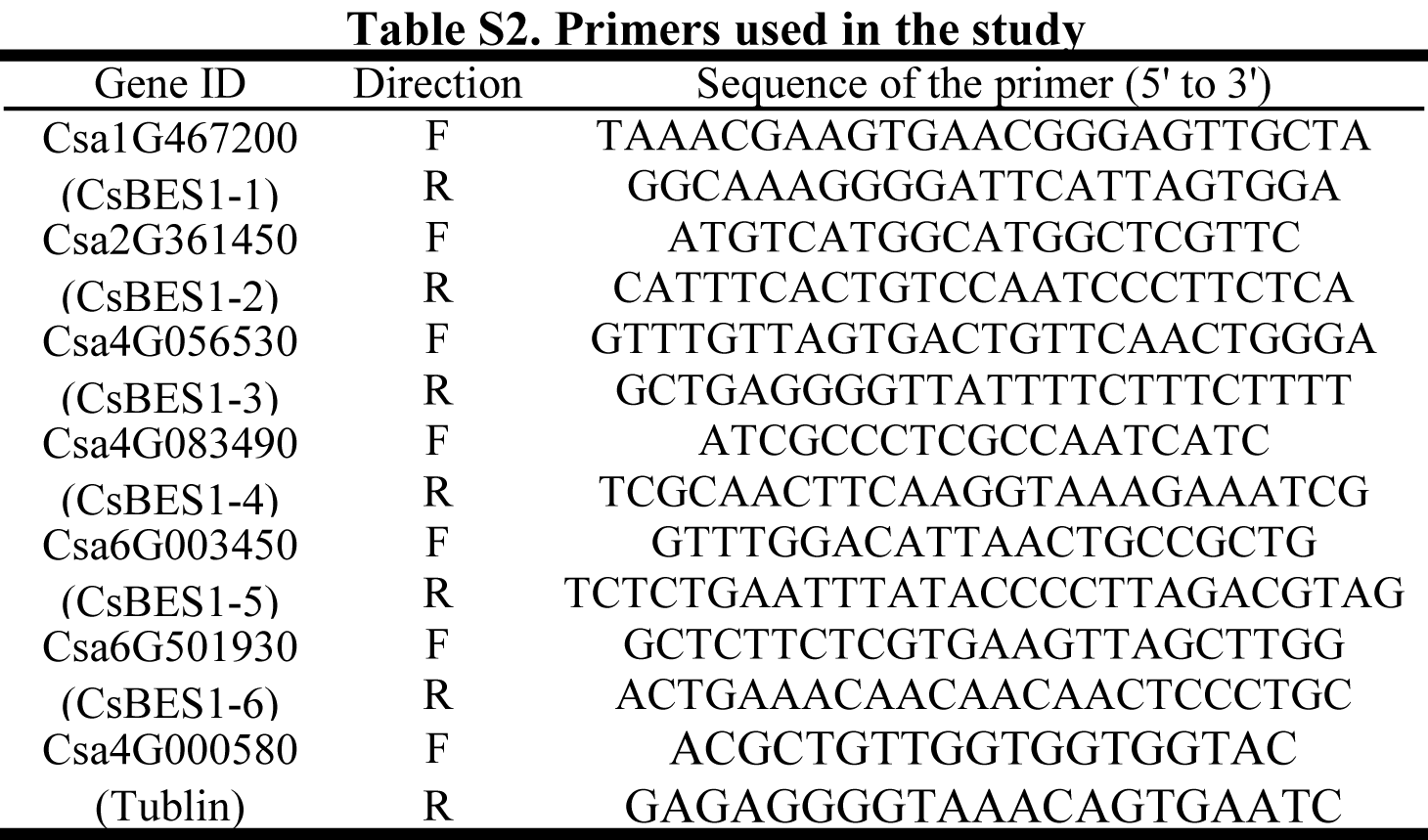

Supplement: Supplementary Table 1 — Primers used in the study. [file Image_4.TIF]
